# The effect of smoking and alcohol consumption on markers of systemic inflammation, immunoglobulin levels and immune response following pneumococcal vaccination in patients with arthritis

**DOI:** 10.1186/ar3923

**Published:** 2012-07-23

**Authors:** Carmen Roseman, Lennart Truedsson, Meliha Crnkic Kapetanovic

**Affiliations:** 1 Department of Clinical Sciences, Section of Rheumatology, Lund University, Kioskgatan 3, Lund SE-221 85, Sweden; 2 Department of Laboratory Medicine, Section of Microbiology, Immunology and Glycobiology, Lund University, Lund SE-221 85, Sweden

## Abstract

**Introduction:**

The purpose of this research was to study the influence of cigarette smoking and alcohol consumption on immune response to heptavalent pneumococcal conjugate vaccine, immunoglobulin levels (Ig) and markers of systemic inflammation in patients with rheumatoid arthritis (RA) or spondylarthropathy (SpA).

**Methods:**

In total, 505 patients were vaccinated. Six pre-specified groups were enrolled: RA on methotrexate (MTX) treatment in some cases other disease-modifying antirheumatic drugs (DMARDs) (I); RA on anti-tumour necrosis factor (TNF) as monotherapy (II); RA on anti-TNF+MTX+ possibly other DMARDs (III); SpA on anti-TNF as monotherapy (IV); SpA on anti-TNF+MTX+ possibly other DMARDs (V); and SpA on nonsteroidal anti-inflammatory drugs (NSAIDs) and/or analgesics (VI). Smoking (pack-years) and alcohol consumption (g/week) were calculated from patient questionnaires. Ig, C-reactive protein (CRP) and erythrocyte sedimentation rate (ESR) were determined at vaccination. IgG antibodies against serotypes 23F and 6B were measured at vaccination and after four to six weeks using standard ELISA. Immune response (ratio between post- and pre-vaccination antibodies; immune response (IR)) and positive immune response (≥2-fold increase in pre-vaccination antibodies; posIR) were calculated.

**Results:**

Eighty-eight patients (17.4%) were current smokers. Smokers had higher CRP and ESR, lower IgG and lower IR for both serotypes (*P *between 0.012 and 0.045). RA patients on MTX who smoked ≥1pack-year had lower posIR for both serotypes (*P *= 0.021; OR 0.29; CI 0.1 to 0.7) compared to never-smokers. Alcohol consumption was associated with lower CRP (*P *= 0.05) and ESR (*P *= 0.003) but did not influence IR or Ig levels.

**Conclusion:**

Smoking predicted impaired immune response to pneumococcal conjugate vaccine in RA patients on MTX. Smokers with arthritis had higher inflammatory markers and lower IgG regardless of diagnosis and treatment. Low to moderate alcohol consumption was related to lower levels of inflammation markers but had no impact on immune response.

**Trial registration:**

EudraCT EU 2007-006539-29 and NCT00828997

## Introduction

Pneumococcal vaccination is recommended to all adults age 65 or older and subjects younger than 65 years with chronic and immunosuppressing conditions. In addition, adult cigarette smokers and immunocompetent persons with underlying conditions, such as alcoholism, should receive this vaccine regardless of age [1]. Cigarette smoking was found to be an independent risk factor for invasive pneumococcal disease in immunocompetent adults younger than 65 years and the risk increased with increased smoking pack-years [2]. It has also been shown that interaction between smoking and certain genetic factors increases risk of rheumatoid arthritis (RA) [3]. Investigation of immune and inflammatory functions in cigarette smokers including levels of immunoglobulins (Ig) and antibody response after vaccination have given conflicting results [4-9]. Excess alcohol is shown to suppress a wide range of immune responses predisposing the host to various infections, and in particular pulmonary ones [9,10]. On the other hand, moderate consumption of alcoholic beverages may enhance immune response [11,12].

The majority of patients with inflammatory arthritis, such as RA or spondylarthropathy (SpA), receiving immunosuppressive treatments with disease-modifying antirheumatic drugs (DMARDs), biologic remedies or long-term systemic steroids are at an increased risk for invasive pneumococcal disease and, therefore, a target group for pneumococcal vaccination. We have demonstrated a diminished antibody response following pneumococcal vaccination (both 23-valent polysaccharide and 7-valent conjugate) in arthritis patients treated with methotrexate (MTX) compared to those receiving anti-tumour necrosis factor (TNF) agents [13,14] and SpA patients not taking any immunosuppressive drugs [14].

The aim of this study was to examine the impact of cigarette smoking and alcohol drinking habits on markers of systemic inflammation, such as C-reactive protein (CRP) and erythrocyte sedimentation rate (ESR), total serum Ig levels and immune response following pneumococcal vaccination using 7-valent pneumococcal conjugate vaccine in patients with RA and SpA treated with different anti-inflammatory remedies.

## Materials and methods

In all, 505 patients with RA (N = 253) and SpA including psoriatic arthritis (N = 252) receiving different anti-inflammatory treatments were vaccinated with a single dose of 7-valent pneumococcal conjugate vaccine (Prevenar®, Whyeth Pharmaceutica, Collegeville, PA, USA) intramuscularly as previously described [14]. All patients were stratified into six pre-specified groups based on diagnosis and treatment. These were: I RA patients on MTX+ in some cases other disease-modifying antirheumatic drugs (DMARDs) (n = 85); II RA on anti-TNF as monotherapy (n = 79); III RA on anti-TNF+MTX+ possibly other DMARDs (n = 89); IV SpA patients on anti-TNF drugs as monotherapy (n = 83); V SpA on anti-TNF drugs+MTX (n = 83) and VI SpA patients on NSAIDs and/or analgesics (n = 86). No changes in anti-rheumatic treatment within four weeks prior to and up to six weeks following vaccination were allowed.

Measurement of IgG, IgA and IgM serum concentrations by nephelometry using Beckman-Coulter reagents on the Immage 800 instrument (Beckman Coulter, Brea, CA, USA) and determination of CRP and ESR were performed at vaccination.

Serotype specific IgG against pneumococcal polysaccharide serotypes 23F and 6B were measured in sera using standard ELISA as described previously [14,15]. Immune response (IR) was defined as the ratio between post- and pre-vaccination antibody levels. GMC (geometric mean concentrations of antibody responses) were calculated. A positive immune response (posIR) was defined as IR ≥2.

All patients were asked to complete a questionnaire regarding smoking and alcohol drinking habits. Original data on smoking status were available for about 80% of participants in the study. Missing data were retrieved through a telephone call from one researcher (CR) to all subjects initially not responding to the questionnaire. Thereby, data on smoking status were available for 502 of 505 study patients. Regarding smoking status, the following groups were defined: current smokers, non-smokers, ex-smokers and ever-smokers. Ever-smokers denote subjects who had smoked for some period of their life or still smoke (ex-smokers + current smokers). Cumulative exposure to cigarette smoking at vaccination was calculated as smoking pack-years. A pack-year corresponds to 20 cigarettes smoked daily for one year. Alcohol consumption was calculated from self-reported data on usage of alcoholic beverages. The number of each alcoholic beverage/week multiplied by average alcohol content in each portion of a drink corresponded to alcohol intake in grams/week [16]. The total alcohol intake/week was calculated as a sum of alcohol content from all alcoholic beverages consumed per week. Data on current alcohol consumption were missing in 24 patients (4.8%). Low to moderate alcohol consumption was defined as total alcohol intake <30 g/day [17].

### Ethical considerations

Consecutive patients fulfilling inclusion criteria were invited to participate in the study. Oral and written information was provided to all subjects who were invited to participate, and written informed consent was obtained from each participant before enrolment. Ethical approval for the vaccination was obtained from the Ethical Review Board at Lund University (file number 97/2007). The study was conducted as an investigator driven clinical trial (EudraCT EU 2007-006539-29 and NCT00828997) and approved by the Swedish medical products agency (MPA; Läkemedelsverket; file number 151: 2007/88047).

### Statistical analyses

Comparisons were done using Chi-squared, Mann-Whitney U test and independent sample T-test and pair sample T-test, when appropriate. Correlations were calculated by Spearman´s correlation coefficient. Univariate analysis of variance (ANCOVA) was used to study the impact of smoking and alcohol consumption on CRP, ESR, Ig levels, pre- and post-vaccination antibody levels for each serotype and IR. Adjustment for multiple comparisons was performed using the Bonferroni corrections method. Effects of smoking and drinking habits on posIR were analysed using binary logistic regression model. A *P*-value of <0.05 was considered significant.

## Results

### Baseline characteristics

Demographic characteristics, smoking and alcohol consumption status, immune responses, levels of inflammatory markers, and Ig levels in the entire study population and in the different treatment groups are summarised in the Table 1.

**Table 1:** Smoking,alcohol drinkingand antibody response following pneumococcal vaccination in entire study population/different groups

	All participants	(I) RA on MTX	(II) RA on anti-TNF as monotheraphy	(III) RA on anti-TNF+ MTX	(IV) SpA on anti-TNF as monotherapy	(V) SpA on anti-TNF+MTX	(VI) SpA on NSAID/analgesics
**Number of patients at vaccination**	**505**	85	79	89	83	83	86

**Females (%)**	**63**	79	87	78	34	53	45

**CRP g/L (mean)**	**6.7**	** 8.9**	**9.4**	**5.9**	**2.9**	**5.1**	**7.7**

**ESR mm/h (mean)**	**21.5**	**28.3**	**28.6**	**24.4**	**11.7**	**15.3 ( 5 15.3**	**19.8**

**Current smokers (%)**	**17**	19	19	18	12	24	12

**Ex-smokers (%)**	**40**	39	38	45	45	39	35

**Ever-smokers (%)**	**57.4**	56.5	57.0	65.2	55.4	60.2	44.2

**Smoking pack-years; mean (SD)**	**19.4 (14)**	22.0 (13)	21.0 (15)	20.4 (14)	16.0 (13)	19.4 (13)	16.8 (13)

**Current alcohol drinking (g/week) mean (SD)**	**9.7 (9)**	13.2 (11)	8.0 (9)	10.4 (13)	12.2 (12)	8.5 (8)	11.8 (9)

**IgG at vaccination (g/L)(SD)**	**10.4 (2.7)**	9.7 (2.5)	11.8 (3.3)	11.0 (3.3)	9.9 (1.9)	9.9 (2.2)	9.9 (2.1)

**IgM at vaccination (g/L)(SD)**	**1.3 (0.8)**	1.1 (0.6)	1.7 (1.0)	1.4 (0.7)	1.3 (0.8)	1.2 (0.7)	1.0 (0.6)

**IgA at vaccination (g/L)(SD)**	**2.7 (1.2)**	2.5 (1.0)	3.3 (1.4)	2.8 (1.2)	2.7 (1.1)	2.4 (1.1)	2.5 (1.1)

**Immune response (IR) for 23F (mean) ^1^**	**3.5**	2.5	3.4	2.2	4.8	3.0	6.6

**Immune response (IR) ^1 ^for 6B (mean)**	**2.3**	1.8	2.7	1.6	3.1	1.8	3.3

**Patients with protective antibody levels for both 23F and 6B 4-6 weeks after vaccination (%)(posIR) ^2^**	**32.9**	21.2	36.7	15.7	50.6	20.5	47.7

**^1^**Immune response = ratio between post- and prevaccination antibody levels.

**^2 ^**Positive immune response (posIR) was defined as IR ≥2.

At vaccination, RA patients on anti-TNF+MTX had significantly lower CRP compared to the other two RA treatment groups (*P *= 0.019 and *P *= 0.009, compared to group I and group II, respectively). IgG, IgA and IgM levels were significantly lower in RA patients on MTX (group I) compared to RA patients on anti-TNF as monotherapy or RA on anti-TNF +MTX (*P *between 0.032 and <0.001; independent sample T-test).

SpA patients on anti-TNF+MTX had lower CRP compared to the other SpA treatment groups (*P *= 0.001 and <0.001, compared to group IV and VI, respectively). No statistically significant differences of Ig levels were seen among groups of patients with SpA.

### The effect of cigarette smoking in the total study population

Of 505 patients enrolled in the study, 290 (57.4%) smoked for some period of their life or still smoke (ever smokers). The total smoking load expressed in number of smoking pack-years (mean; SD; range) was 19.3 (14; 0 to 65). In total, 212 patients (42%) had never smoked and no data on smoking status were available in 3 patients (0.6%). Two hundred, two patients (40%) had previously smoked but quit smoking before the study entry (ex-smokers). Mean (SD; range) of smoking pack-years in this group was 16.6 (13; 1 to 65).

In all, 88 patients (17.4%) of the population studied were current smokers at the time of vaccination. Mean (SD; range) number of cigarettes per day was 10.1 (6.4; 0 to 30). Mean (SD; range) number of smoking pack-years was 24.5 (13; 0 to 57). Of all current smokers, 64 individuals (72.7%) were females and 24 (27.3%) males (*P *= 0.029).

Differences in measured markers of inflammation, IgG levels and GMC of immune responses for each serotype between non-smokers, ex-smokers and current smokers are given in Table 2.

**Table 2:** Markers of inflammation, Ig and immune response according to smoking status in total study population

	Current smokers (N = 88)	Non-smokers (never-smoked) (N = 212)	Ex-smoker (N = 202)
**Smoking pack-years (mean; SD)**	24.5 (13)*	---	16.6 (13)*

**CRP g/L (mean) (SD)**	10.1 (25)**	5.9 (10)**	5.8 (11)**

**ESR mm/hour (mean) (SD)**	25.6 (22)***	19.8 (17)***	21.4 (19)

**IgG g/L (mean) (SD)**	9.9 (3) #	10.6 (2)	10.4 (3)

**IgA g/L (mean) (SD)**	2.6 (1)	2.7 (1)	2.7 (1)

**IgM g/L (mean) (SD)**	1.2 (0.6)	1.3 (0.9)	1.3 (0.7)

**GMC¤ immune response 23F**	2.6 ##	3.7	3.3

**GMC¤ immune response 6B**	1.8 ###	2.4	2.2

GMC¤ = geometric mean concentration of immune response

*P <0.001 compared to ex-smokers (independent sample T-test)

** *P *= 0.04 compared to non smokers (never-smoked); p = 0.045 compared to ex-smokers (independent sample T-test)

*** *P *= 0.023 compared to non-smokers (never-smoked); independent sample T-test

#*P *= 0.028 compared to non-smokers (never-smoked); independent sample T-test

## *P *= 0.028 compared to non-smokers (never-smoked); Mann-Whitney U test

### *P *= 0.0042 compared to non-smokers (never-smoked); Mann-Whitney U test

Current smokers had significantly higher CRP and ESR compared to non-smokers (Table 2). Number of pack-years of smoking was significantly correlated with both CRP (Spearman's rho = 0.152; *P *= 0.001) and ESR (Spearman's rho = 0.159, *P *= 0.001). The findings persisted in univariate ANCOVA with age, gender, number of tender and swollen joints in 28-joint count index, MTX, anti-TNF treatment, prednisolone and alcohol consumption included in the analysis. Smoking had a significant impact on both CRP (*P *= 0.008) and ESR (*P *= 0.037).

At the time of vaccination, current smokers had significantly lower total IgG levels compared to non-smokers but IgA and IgM levels did not differ significantly. Both daily cigarette number at the time of vaccination and number of pack-years were inversely correlated with IgG levels (Spearman's rho -0.132 (*P *= 0.003) and -0.130 (*P *= 0.006), respectively). These findings were confirmed by ANCOVA with age, gender, DAS, MTX, anti-TNF prednisolone treatment and alcohol consumption as covariates.

GMC of antibody responses, that is, the ratio between post- and pre-vaccination antibody levels, were significantly higher in non-smokers for both serotypes (Table 2). This was not corroborated by univariate ANCOVA after adjustment for demographic, disease and treatment characteristics. Neither did smoking status predict a posIR for each serotype separately nor both 23F and 6B together. Number of pack-years did not correlate with antibody levels or IR and did not predict a posIR for either serotype or both serotypes together.

### Effect of cigarette smoking within different diagnostic and treatment groups

The percentage of current smokers in different treatments groups were: 19, 18, 19, 12, 24 and 12% (Table 1). The proportion of current smokers did not differ significantly between the groups of patients with RA. Among patients with SpA more patients on anti-TNF+MTX were smokers (*P *= 0.05 and 0.04, compared to group 4 and 6, respectively).

### Group I (RA on MTX)

**Figure 1:**
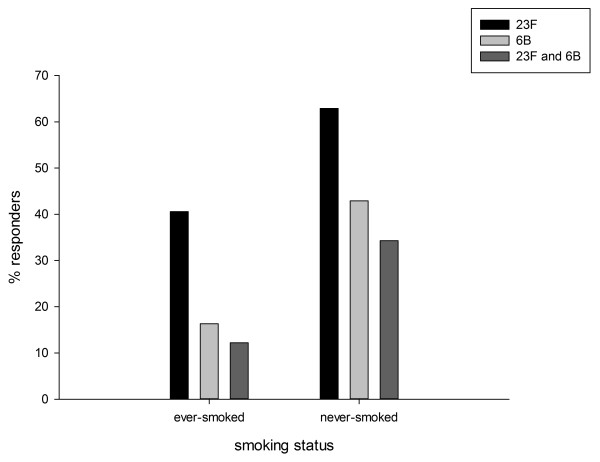
**Responders(%) to 23F, 6B and both pneumococcal serotypes in methotrexate treated RA patients according to smoking status**. Responders i.e. patients with positive immune response defined as post-vaccination antibody levels ≥2 times pre-vaccination antibody levels. Ever smoked i.e. previous/current smokers

In RA patients on MTX, current smokers had significantly higher CRP and ESR. This was also demonstrated using univariate ANCOVA, where age, gender, number of tender and swollen joints in 28-joint count index were included in the analysis (*P *= 0.023 for CRP and *P *= 0.023 for ESR). However, total IgG, IgA and IgM levels did not differ between smokers and non-smokers in this treatment group. Ever-smokers among RA patients on MTX had a lower proportion of responders to vaccination for both serotypes (*P *= 0.025; OR 0.21; 95% CI 0.05 to 0.81) compared to never-smokers (Figure 1). Also, higher pack-years were associated with lower posIR (*P *= 0.046; OR 0.93 95% CI 0.86 to 0.99) after adjustment for age, sex, CRP, counts of affected joints (tender joint count (TJC) and swollen joint count (SJC)) and pre-vaccination antibody levels (Table 3).

**Table 3:** Impact of smoking on positive immune response forpneumococcal serotypes(23F and 6B) in RA patients on methotrexate

	B	*P*-value	Exp(B)	95% CI	
				
				Lower	Upper
**Smoking (pack-years)**	**-0.07**	**0.046**	**0.93**	**0.86**	**0.99**

Age (years)	-0.01	0.724	0.99	0.95	1.03

Gender	0.85	0.341	2.34	0.41	13.43

CRP	-0.03	0.639	0.98	0.88	1.08

SJC28*	-0.09	0.502	0.91	0.69	1.20

TJC28*	0.00	1.000	1.0	0.76	1.32

Pre-vaccination antibody levels for serotype 23F	-0.42	0.090	0.66	0.41	1.07

**Smoking (ever)**	**-1.58**	0.025	**0.21**	**0.05**	**0.81**

*swollen and tender joint count in 28-joint count index

Two different logistic regression models were created analyzing smoking (pack-years) and smoking (ever) variable, respectively adjusted for the same covariates.

### Groups II to VI

Smoking habits had no effect on acute phase reactants, Ig levels, pre- and post-vaccination antibody levels, IR or posIR against pneumococcal serotypes 6B or 23F in any of the other treatment groups.

### Effect of alcohol drinking in the total study population

Of the participating 505 patients, 148 (29.3%) reported no or sporadic alcohol consumption in very small amounts, while 333 (65.9%) patients reported alcohol consumption on a regular basis. Median (range) consumption among patients using alcoholic beverages was 70.8 (5.3 to 758) g/week. The majority of patients participating in the study reported low to moderate alcohol use corresponding to ≤30 g 100% ethanol daily. Alcohol consumption was more common in men (*P *<0.001).

Compared to non-drinkers, patients regularly consuming alcohol showed lower levels of CRP and ESR (*P *= 0.007 and *P *<0.001; T-test) also remaining after univariate ANCOVA, including age, gender, number of tender and swollen joints in 28-joint count index, and after adjustment for multiple comparisons (*P *= 0.05 and *P *= 0.003, for CRP and ESR, respectively).

However, serum Ig levels, GMC of antibody responses or posIR were not influenced by the reported alcohol consumption.

### Effect of alcohol drinking within different diagnostic and treatment groups

Within each treatment group, no significant differences in CRP, ESR, Ig levels, GMC of antibody responses or pos IR were detected among persons not consuming alcoholic beverages compared to those drinking alcohol.

## Discussion

In this study, we report the impact of patient reported smoking and alcohol drinking habits on markers of systemic inflammation (CRP and ESR), serum level of Ig and immune response after standardised antigen challenge (pneumococcal vaccination using heptavalent conjugate vaccine) in patients with established RA and spondylarthropathy treated with different anti-inflammatory remedies.

A main finding is that smokers in this study had higher acute phase reactants, such as CRP and ESR, compared to non-smokers regardless of anti-rheumatic treatment. Our results are in accordance with some other previously published studies reporting the impact of smoking on several markers of systemic inflammation [8,18-20]. Patients with RA and SpA are known to have an increased risk of cardiovascular disease, probably mediated through the inflammatory nature of these diseases but smoking may further increase the risk.

Another important finding is that, in the entire population of arthritis patients participating in this study, current smokers had lower serum levels of IgG compared with non-smokers regardless of diagnosis or ongoing anti-rheumatic treatment. Both higher numbers of cigarettes smoked daily and total smoking load expressed as number of pack-years were associated with lower IgG levels. These results are in accordance with earlier reports on 10 to 20% reduced Ig concentrations in smokers [8] and more recent findings showing lower Ig concentrations in healthy individuals smoking for more than 10 years [5].

Total IgG, IgM and IgA levels were significantly lower in RA patients treated with MTX compared to RA patients treated with anti-TNF as monotherapy or anti-TNF combined with MTX. The mechanisms of action of MTX are complex and not entirely known [21] but probably include the effect on B-cells as well. Decreased numbers and subsets of memory B-cells have recently been reported in patients with RA compared with age matched controls [22]. Among RA patients, those with MTX treatment had significantly lower B-cell numbers compared to RA patients treated with anti-TNF and MTX in combination [21,22]. We found no differences in Ig levels between SpA treatment groups. On the other hand, patients with SpA only treated with MTX were not included in this study and the influence of RA on Ig levels cannot be entirely ruled out. One may speculate that RA patients on MTX monotherapy may have a less severe disease and thus a more profound immunological impact of MTX on their immune system compared to RA patients requiring additional or other remedies for disease control.

When the effects of current smoking on immune response following pneumococcal vaccination was analysed in the entire study population, we detected significantly lower antibody responses in current smokers. However, these differences between smokers and non-smokers were not seen after correcting for treatment group and disease characteristics. Also, the results are in contrast to a reported increased level of pneumococcal antibodies in smokers [2]. The contrasting findings reported may reflect differences in study population since the previous study comprised a random sample of elderly inhabitants aged 64 to 97 years probably not treated with immunosuppressive drugs. Since very few women enrolled in that study were smokers, only men were included in analysis of smoking effects on antibody response. Interestingly, compared to men, significantly more women in our study were smokers. The higher proportion of smokers among women in our study could be explained by opposite trends in the smoking behaviour between men and women recognised later in the 20^th ^century in the industrialised countries when smoking became more common among women but decreased in men [23]. A pathogenetic impact of smoking on RA disease possibly with greater penetrance in women cannot be ruled out [3]. The immunogenicity between pneumococcal serotypes differs significantly and since antibody response to different serotypes were analysed in the mentioned study [4], our results cannot be directly compared. Serotypes 23F and 6B are reported to be associated with invasive pneumococcal diseases in Sweden, which was the main reason for choosing these particular two serotypes in the present study [24]. Analyzing all seven serotypes in the vaccine would result in a more exact and probably also more reliable estimation of antibody responses in the study population. However, we assume that the underlying mechanisms responsible for effects of smoking and drinking on antibody response would be similar in all serotypes.

When the effect of smoking on immune response was analysed in the separate treatment groups, both ever smoking and number of pack-years were associated with impaired antibody response only in RA patients on MTX monotherapy. We have previously reported that this group has the lowest IR and posIR following pneumococcal vaccination [13,14]. Additionally, we now find both current smoking and number of pack-years to predict decreased immune response within this group after adjustment for demographic and relevant disease characteristics.

In contrast to smoking, alcohol consumption was associated with lower levels of CRP and ESR in the entire study population. The vast majority of patients participating in this study reported low to moderate alcohol consumption with median weekly consumption of 71 g. Our results are in line with other reports on anti-inflammatory and cardioprotective effects of moderate alcohol consumption [11,12,17].

In summary, in the present study we confirm that smoking can influence immune response. The most pronounced effect was seen in RA patients treated with MTX as monotherapy where smoking was identified as a predictor of diminished immune response following pneumococcal vaccination. Both smoking and alcohol consumption had an impact on systemic inflammation: smoking being associated with higher and alcohol with lower level of inflammatory markers. Our results contribute to the growing evidence of negative effects of smoking and possible advantages of moderate alcohol drinking in patients with established arthritis.

## Conclusions

Smoking predicted impaired immune response to pneumococcal conjugate vaccine in RA patients on MTX. Smokers with arthritis had higher inflammatory markers and lower IgG regardless of diagnosis and treatment. Low to moderate alcohol consumption was related to lower levels of inflammation markers but had no impact on immune response.

## Abbreviations

CRP: C-reactive protein; DMARDs: disease modifying anti-rheumatic drugs; ESR: erythrocyte sedimentation levels; GMC: geometric mean concentration; Ig: immunoglobulin levels; IR: immune response; MTX: methotrexate; NSAIDS: nonsteroidal anti-inflammatory drugs; posIR: positive immune response; RA: rheumatoid arthritis; SJC: swollen joint count; SpA: spondylarthropathy; TJC: tender joint count; TNF: tumour necrosis factor.

## Competing interests

The authors declare that they have no competing interests.

## Authors' contributions

CR participated in the design of the study, performed the statistical analysis and prepared the manuscript. LT and MCK conceived of the study, participated in its design and coordination, and helped to draft the manuscript. All authors read and approved the final manuscript.
